# Supplementary material for: Cavity-enhanced microwave readout of a solid-state spin sensor
Source: Nat Commun. 2021 Mar 1;12:1357. doi: 10.1038/s41467-021-21256-7 (PMC7921108; doi:10.1038/s41467-021-21256-7)
Supplement: Supplementary file 1 — Supplementary Information [file 41467_2021_21256_MOESM1_ESM.pdf]

Supplementary Information:
Cavity-enhanced microwave readout of a solid-state spin sensor

I. SUPPLEMENTARY NOTE 1: SENSITIVITY AND FIDELITY

A. Johnson noise sensitivity limit

Johnson-Nyquist (thermal) noise degrades the microwave signal following interaction with the composite cavity. The composite cavity produces Johnson-Nyquist noise $V_{\mathrm{JN}} = \sqrt{4k_B T R_s \Delta f}$, where k_B is the Boltzmann constant, T is the cavity's temperature, R_s is the equivalent resistance of the composite cavity, and Δf is the single-sided measurement bandwidth. Assuming that the cavity is impedance matched to the termination resistance $R = 50\ \Omega$, this produces a noise voltage $\sqrt{k_B T R \Delta f}$ at the measurement device. The Johnson-Nyquist-limited sensitivity is then given by

$$\eta_{\mathrm{JN}} = \frac{\sqrt{k_B T R}}{F_1 \cdot F_2 \cdot \left[\frac{d(\mathrm{Im}[\Gamma]V_{\mathrm{RMS}})}{dB_0}\right]_{\max}}, \tag{1}$$

where $\mathrm{Im}[\Gamma]V_{\mathrm{RMS}}$ is the quadrature component of the reflected RMS MW voltage, B_0 is the magnetic field, and F_1 and F_2 are factors of order unity depending on details of the signal and the processing architecture respectively. For scenarios where the phase of the signal is assumed to be known (as in this work), $F_1 = \sqrt{2}$. For architectures where all signal is isolated into the quadrature channel of the mixer (as in this work), we expect $F_2 = \sqrt{2}$. At 10 dBm of applied MW power, we then estimate $\frac{d(\mathrm{Im}[\Gamma]V_{\mathrm{RMS}})}{dB_0} \approx 0.05$ V/G (Supplementary Fig. 3), yielding $\eta_{\mathrm{JN}} \approx 0.5$ pT/$\sqrt{\mathrm{Hz}}$.

B. Spin-projection sensitivity limit

The spin-projection-limited magnetic sensitivity is given by [1]

$$\eta_{\mathrm{sp}} \approx \frac{\hbar}{g_e \mu_B} \frac{1}{\sqrt{N\tau}}, \tag{2}$$

where τ is the free precession time. For a Ramsey measurement scheme, it is nearly optimal to choose a precession time equal to the dephasing time, T_2^* [2]. Although there is no explicit precession time in a CW measurement scheme, we make the substitution $\tau = T_2^*$ in the above equation as a crude estimate of the effective precession time. For our measurement scheme, two additional factors must be considered, each of which degrades the spin-projection-noise-limited sensitivity. First, the projection of the magnetic field on each NV$^-$ orientation is $B_0/\sqrt{3}$, so that the magnetic sensitivity is a factor $\sqrt{3}$ larger than naïvely expected. Second, the finite time required (on average) to optically initialize a spin, T_1^{op}, produces an effective duty cycle for the measurement of τ/T_1^{op} (assuming $T_1^{\mathrm{op}} \gg \tau$). Again making the substitution $\tau = T_2^*$, this effects a degredation of the sensitivity by the factor $\sqrt{T_1^{\mathrm{op}}/T_2^*}$. With these modifications, the appropriate spin-projection-limited sensitivity for this measurement scheme (in the absence of all technical noise) is approximately given by

$$\eta_{\mathrm{sp}}^{\mathrm{op}} \approx \sqrt{3}\frac{\hbar}{g_e \mu_B} \frac{1}{\sqrt{N T_2^*}} \sqrt{\frac{T_1^{\mathrm{op}}}{T_2^*}}. \tag{3}$$

To compute T_1^{op}, we assume that M green photons are required, on average, to polarize one NV$^-$ center. We expect $M > 1$ due to the limited asymmetry of decay to $|m_s = 0\rangle$ through the intersystem crossing and because green photons may be "wasted" through radiative decay or absorption by substitutional nitrogen, NV^0, and other defects. We crudely guess the value of M is between between 1 and 10. We calculate T_1^{op} as

$$T_1^{\mathrm{op}} = M \frac{N}{R_{\mathrm{pho}}} \tag{4}$$

where R_{pho} is the number of photons per second applied to the NV ensemble. For $M = 3$, assuming 12 W of 532 nm light and $N = 1.4 \times 10^{15}$ polarized NV$^-$ centers, we have $T_1^{\mathrm{op}} = 130$ μs and $\eta_{\mathrm{sp}}^{\mathrm{op}} = 75$ fT/$\sqrt{\mathrm{Hz}}$.

C. Microwave photon noise limit to readout fidelity

It is instructive to calculate the expected inverse readout fidelity $\sigma_R = 1/\mathcal{F}$ in the absence of thermal (Johnson-Nyquist) and other technical noise (e.g. phase noise). The inverse readout fidelity (equal to the factor over spin projection noise) is

$$\sigma_R = \sqrt{1 + \frac{1}{C^2 n_{\text{avg}}}}, \tag{5}$$

where C is the readout contrast (fringe visibility) and n_{avg} represents the average number of MW photons collected per NV$^-$ per measurement. At 10 dBm of applied MW power, the composite cavity reflects a maximum -2.4 dBm, which corresponds to 3.0×10^{20} MW photons/second. We assume $N = 1.4 \times 10^{15}$, $T_1^{\text{op}} = 130$ µs, and $C = 1$. This crude estimate gives $n_{\text{avg}} = 28$, resulting in $\sigma_R = 1.017$ and $\mathcal{F} = .983$. Employing instead $T_2 = 8$ µs results in $\sigma_R = 1.254$ and $\mathcal{F} = .798$. Employing $T_2^* = 40$ ns yields $\sigma_R = 10.7$ and $\mathcal{F} = 0.093$. For any of these timescales, the shot-noise-limited sensitivity $\eta_{\text{sh}} = \sigma_R \eta_{\text{sp}}^{\text{op}}$ is substantially less than the Johnson-Nyquist-limited sensitivity, so that the readout is not limited by MW photon shot noise.

II. SUPPLEMENTARY NOTE 2: SPIN ENSEMBLE SUSCEPTIBILITY

A. Static susceptibility

Optical pumping preferentially populates the $|m_s = 0\rangle$ electronic ground state which is separated from the $|m_s = \pm 1\rangle$ states by the zero-field splitting D. In the vicinity of the resonance $\omega_s = 2\pi D + \gamma B_z$ between the $|m_s = 0\rangle$ and $|m_s = +1\rangle$ states under an applied magnetic field $\mathbf{B}_0$, where B_z is the projection of $\mathbf{B}_0$ onto the quantization axis set by the NV orientation, we consider the response of an effective two-level system. The static susceptibility is

$$\chi_0 = \frac{\mu_0 (g\mu_B)^2}{\hbar \omega_s} \frac{N_0 - N_{+1}}{V} \tag{6}$$

where N_{m_s} is the number of spins in the m_s state, and V the volume. We define the number of polarized spins used in the main text as $N = N_0 - N_{+1}$. The susceptibility of the diamond with volume V_{dia} evaluates to $\chi_0^{\text{dia}} = 1.3 \times 10^{-5}$, whereas the susceptibility of the whole composite cavity (with volume V_{cav}) is $\chi_0 = 3.5 \times 10^{-7}$.

B. AC susceptibility

To understand the spin-ensemble susceptibility, consider the response of the magnetization $\mathbf{M}$ to a driving field $\mathbf{H}$. The complex magnetic susceptibility $\chi = \chi' - j\chi''$ characterizes the response, and obeys $\mathbf{M} = \chi \mathbf{H}$. Evaluating the steady-state solution of the Bloch equations under application of a linearly polarized oscillating field $H(t) = H_1 \cos(\omega t)$, the real and imaginary components of χ [3],

$$\chi' = -\frac{1}{2}\chi_0 \frac{\omega_s(\omega_d - \omega_s)T_2^2}{1 + (\omega_d - \omega_s)^2 T_2^2 + \left(\frac{\gamma B_1^\perp}{2}\right)^2 T_1^{\text{op}} T_2} \tag{7}$$

and

$$\chi'' = \frac{1}{2}\chi_0 \frac{\omega_s T_2}{1 + (\omega_d - \omega_s)^2 T_2^2 + \left(\frac{\gamma B_1^\perp}{2}\right)^2 T_1^{\text{op}} T_2}, \tag{8}$$

characterize the dispersion and absorption, respectively. Here, $B_1^\perp = \mathfrak{n}_\perp B_1$ is the projection of the driving field $\mu_0 H_1 = B_1$ onto the plane perpendicular to each of the NV axes (consequently $\mathfrak{n}_\perp = \sqrt{\frac{2}{3}}$), T_1^{op} is the optical pumping relaxation time (in analogy to the thermalization time T_1 for an NMR system), T_2 is the decoherence time, and ω_d is

the drive frequency. To aid in deriving reflection and transmission coefficients, we rewrite the complex susceptibility into the compact form

$$\chi = -\frac{\chi_0 \omega_s T_2}{2} \frac{j}{1 + j(\omega_d - \omega_s)T_2 + \dfrac{\left(\gamma B_1^{\perp}/2\right)^2 T_1^{\mathrm{op}} T_2}{1 - j(\omega_d - \omega_s)T_2}}. \tag{9}$$

III. SUPPLEMENTARY NOTE 3: COMPOSITE RESONATOR MODELS

In this section, we analyze the electromagnetic fields and equivalent circuit models of the composite dielectric resonator [4]. Initially considering the case where only the input loop couples to the resonator, neglecting the output coupling loop, we study the electromagnetic fields of the resonator and coupling loop. From here we derive multiple equivalent circuit models, with circuit component values determined from analysis of the fields of the dielectric resonator's mode of interest, shown in Supplementary Fig. 1. After incorporating the spin-ensemble response and output coupling loop, we obtain reflection and transmission coefficients which describe the full response of the system presented in this work.

A. Electromagnetic field and equivalent circuit representations

We can understand the electric and magnetic fields of the resonator by analyzing a cylindrical dielectric waveguide that has magnetic walls and is open on both ends [5]. For a dielectric resonator of height L, radius a, and relative permittivity ϵ_r, the fields in the resonator's $\mathrm{TE}_{01\delta}$ mode (Supplementary Fig. 1a) are, in cylindrical coordinates,

$$H_z = H_1 J_0(k_c \rho) f(\tilde{z}), \tag{10}$$

$$H_\rho = -\frac{j\beta}{k_c} H_1 J_1(k_c \rho) f'(\tilde{z}), \tag{11}$$

and

$$E_\phi = -\frac{j\omega\mu_0}{k_c} H_1 J_1(k_c \rho) f(\tilde{z}), \tag{12}$$

where we have taken the fields to have time dependence $e^{j\omega t}$. Here $J_n(x)$ are Bessel functions of the first kind of order n, H_1 is the field amplitude, ω is the oscillation frequency, and $f(\tilde{z})$ gives the longitudinal dependence,

$$f(\tilde{z}) = \begin{cases} \cos \beta z, & |z| < \frac{L}{2} \\ \cos \frac{\beta L}{2} e^{-\alpha(|z| - L/2)}, & |z| > \frac{L}{2} \end{cases}. \tag{13}$$

Here, $k_c = \frac{x_{01}}{a}$, where x_{01} is the first zero of the zeroth-order Bessel function, $\beta = \sqrt{\epsilon_r k_0^2 - k_c^2}$ is the propagation constant in the dielectric region, and $\alpha = \sqrt{k_c^2 - k_0^2}$ is the attenuation constant outside the resonator, with $k_0 = \omega/c$ the free-space wavenumber.

From the fields, we now want to compute the impedance of the dielectric resonator in terms of its geometrical and material properties. We start by computing the total power delivered to the resonator,

$$P = P_d + j2\omega(W_m - W_e), \tag{14}$$

where

$$P_d = \frac{1}{2}\int_{\text{resonator}} dv \sigma |E|^2 \tag{15}$$

is the time-averaged power dissipated in the dielectric, and

$$W_m = \int_{\text{all space}} dv \frac{1}{4}\mu |H|^2 \tag{16}$$

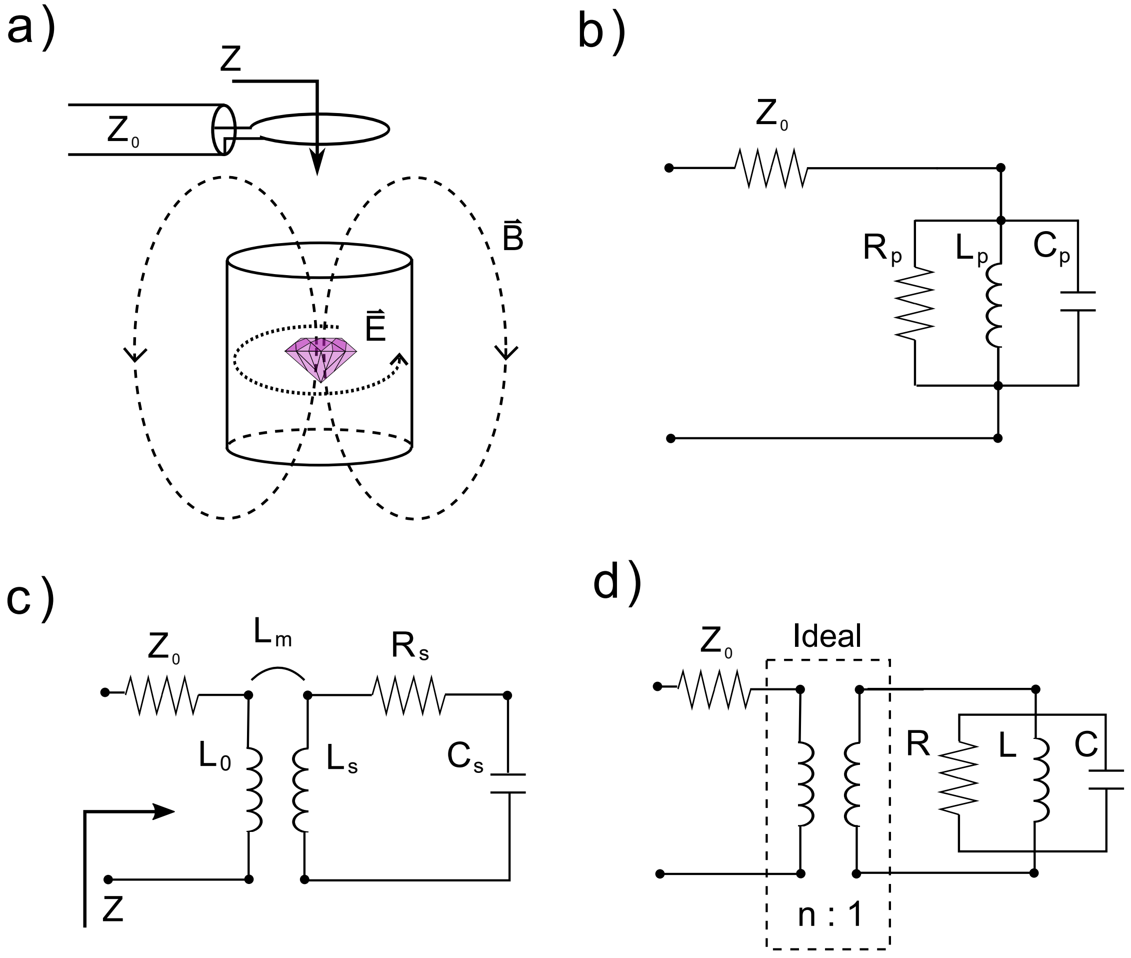

Supplementary Fig. 1. **Electromagnetic field and equivalent circuit representations of the dielectric resonator** a) Illustration of physical resonator and $TE_{01\delta}$ mode with impedance Z looking in to coupling loop. b) Parallel RLC circuit as extracted from electromagnetic field model. Coupling is implicitly contained within calculated circuit components. c) Circuit in b) transformed as series RLC circuit. Coupling represented by mutual inductance L_m between loop inductance L_0 and series resonator inductance L_s. d) Mutual inductance coupling represented as ideal transformer with ratio $n:1$ and lumped element components Supplementary Eq. (32).

and

$$W_e = \int_{\text{all space}} dv \frac{1}{4}\epsilon |E|^2 \tag{17}$$

are the respective magnetic and electric time-averaged energies stored in the resonator (and dv is a volume element). The impedance Z looking into the loop is related to the complex conjugate of the power P^* delivered to the resonator through

$$\frac{1}{Z} = \frac{2P^*}{|V|^2}, \tag{18}$$

where the electric potential V induced in the loop due to the fields of the resonator (arising from the changing magnetic flux through the loop) is

$$V = -\oint_l \mathbf{E} \cdot d\mathbf{l} = j\omega\mu_0 \int_S \mathbf{H} \cdot d\mathbf{S}. \tag{19}$$

The line integral of the electric field is taken around the loop with line element $d\mathbf{l}$ and total length l, and the surface integral incorporates the magnetic field through the loop of area S and area element $d\mathbf{S}$ oriented normal to the plane of the loop.

The impedance looking into the loop takes the form of a parallel RLC circuit $1/Z = 1/R_p + (1/j\omega L_p + j\omega C_p)$. The equivalent parallel circuit resistance, inductance, and capacitance,

$$R_p = \frac{2S^2 x_{01}^2 e^{-2\alpha d} \cos^2 \frac{\beta L}{2}}{\pi a^4 L\omega\epsilon_0\epsilon_r \tan\delta_l [J_1(x_{01})]^2 \left[1 + \frac{1}{\beta L}\sin\beta L\right]} \tag{20}$$

$$L_p = \frac{\mu_0 S^2}{\pi\ a^2 L} \frac{e^{-2\alpha d}\cos^2\frac{\beta L}{2}}{[J_1(x_{01})]^2 \left[\left(1 + \left(\frac{\beta}{k_c}\right)^2\right)\left(\frac{1}{2} + \frac{1}{\alpha L}\cos^2\frac{\beta L}{2}\right) + \left(1 - \left(\frac{\beta}{k_c}\right)^2\right)\frac{1}{2\beta L}\sin\beta L\right]} \tag{21}$$

$$C_p = \frac{\pi\ a^4 L\epsilon_0\epsilon_r}{S^2 x_{01}^2} \frac{[J_1(x_{01})]^2 \left[\frac{1}{2}\left(1 + \frac{1}{\beta L}\sin\beta L\right) + \frac{1}{\epsilon_r \alpha L}\cos^2\frac{\beta L}{2}\right]}{e^{-2\alpha d}\cos^2\frac{\beta L}{2}}, \tag{22}$$

can be obtained using Supplementary Eq. (10)-(19), with d the distance from the top of the cylinder to the loop, and $\tan\delta_l$ the loss tangent of the dielectric. These values implicitly contain the mutual inductance L_m between the loop and the resonator. The parallel circuit in Supplementary Fig. 1b can be transformed into an equivalent series RLC circuit representation [6] with explicit coupling between the loop and resonator (Supplementary Fig. 1c). The equivalent series lumped-element parameters

$$L_s = \frac{L_m^2}{L_p}, \tag{23}$$

$$C_s = \frac{C_p L_p^2}{L_m^2}, \tag{24}$$

$$R_s = \frac{L_m^2}{C_p L_p R_p}. \tag{25}$$

represent the bare resonator apart from the coupling loop. We can calculate the inductance L_s of the bare resonator directly from the magnetic energy and current in the resonator. The displacement current density $\mathbf{J}_d = \epsilon\partial\mathbf{E}/\partial t$ gives the current

$$I = \int \mathbf{J}_d \cdot d\mathbf{A} = \frac{2\epsilon_r k_0^2 H_1 \sin\frac{\beta L}{2}}{k_c^2\beta}. \tag{26}$$

Using the average magnetic energy $W_m = L_s I^2/4$ then gives the resonator inductance

$$L_s = \frac{\mu_0\pi\ a^2 L k_c^4\beta^2}{4\epsilon_r^2 k_0^4} \frac{[J_1(x_{01})]^2}{\sin^2\frac{\beta L}{2}} \left[\left(1 + \left(\frac{\beta}{k_c}\right)^2\right)\left(\frac{1}{2} + \frac{1}{\alpha L}\cos^2\frac{\beta L}{2}\right) + \left(1 - \left(\frac{\beta}{k_c}\right)^2\right)\frac{1}{2\beta L}\sin\beta L\right] \tag{27}$$

and we find, as expected, that L_s is independent of the coupling loop area or distance above the resonator. We then find the mutual inductance

$$L_m = \frac{\mu_0 S\beta k_c^2}{2\epsilon_r k_0^2}\cot\left(\frac{\beta L}{2}\right)e^{-\alpha d} \tag{28}$$

which we can use to find the resonator series equivalent capacitance and resistance. We calculate

$$L_s = 3.88\ \text{nH},\ C_s = 0.776\ \text{pF}, \text{and}\ R_s = 3.17\ \text{m}\Omega. \tag{29}$$

Using HFSS we obtain the equivalent series RLC circuit component values of the bare cavity (i.e. the resonator cavity and diamond system in the absence of any spin polarization by the laser) which are computed using the following procedure: first, finite element modeling software (Ansys HFSS) calculates the $\mathbf{E}$ and $\mathbf{B}$ fields for a given stored energy in the cavity; next, the Ampere-Maxwell law is applied to determine the RMS displacement current I_D^{RMS}; third, the stored magnetic energy is set equal to $\frac{1}{2}L_s(I_D^{\text{RMS}})^2$ to determine the value of L_s; and finally, the capacitance is determined from the resonant frequency and the inductance, with $C_s = \frac{1}{\omega_c^2 L_s}$. The series resistance R_s

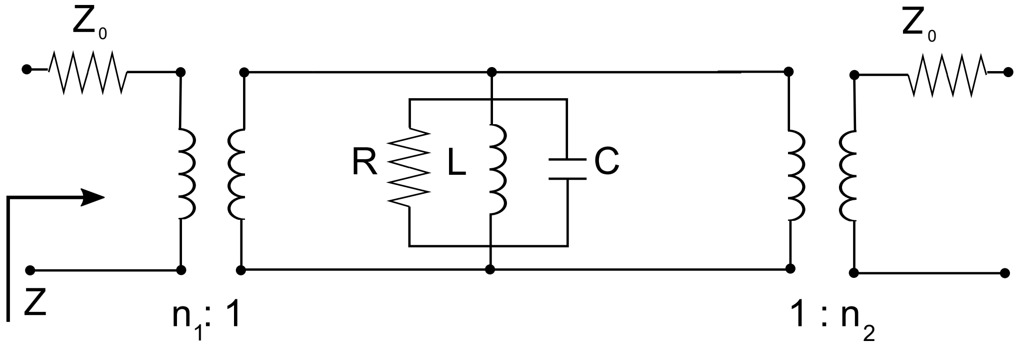

Supplementary Fig. 2. **Full lumped element circuit model** Illustration of the ideal-transformer-coupled parallel RLC circuit with out- and input couplings. The lumped element components are Supplementary Eq. (32) and the input and output coupling turns ratios are n_1 and n_2 respectively.

is computed by first experimentally measuring the composite cavity's unloaded quality factor Q_0, with $R_s = \frac{1}{Q_0}\sqrt{\frac{L_s}{C_s}}$. This procedure gives

$$L_s = 3.75 \text{ nH}, \, C_s = 0.803 \text{ pF}, \text{and}\, R_s = 3.15 \text{ m}\Omega. \tag{30}$$

These results quantitatively agree with the analytical model of the electromagnetic fields of the resonator $\text{TE}_{01\delta}$ mode described above to within 3.5%.

For ease of analysis a further transformation can then be made, where the parallel RLC circuit described by the lumped elements in Supplementary Eq. (20) - (22) is replaced by an ideal-transformer-coupled parallel RLC circuit with lumped elements constructed from the series equivalent RLC circuit (Supplementary Fig. 1d). The benefit of this construction is three fold: far from resonance the correct phase relationship between the voltage and current is maintained, the lumped element components remain independent from the coupling which allows for introducing an arbitrary number of additional loop couplings without loss of generality, and, finally, computation of the circuit reflection (and transmission in the case of added output couplings) coefficient becomes a trivial exercise in classical circuit theory. We rewrite Supplementary Eq. (20) - (22) in terms of the series equivalent circuit values and an effective turns ratio $n : 1$, where we have followed traditional electrical engineering convention and defined n as the ratio of primary-to-secondary turns of an ideal transformer. The effective turns ratio is given by

$$n = \frac{L_m}{L_s} = \frac{L_p}{L_m}. \tag{31}$$

The modification of the series inductance as a result of this transformation is given by $L_s' = L_s - n^2 L_0$. Here, however, we have $n^2 L_0 << L_s$, and thus we set $L_s' \approx L_s$ [6]. The ideal-transformer-coupled parallel RLC lumped element circuit components are

$$L = L_s \tag{32}$$

$$C = C_s \tag{33}$$

$$R = \frac{\omega_c^2 L_s^2}{R_s}. \tag{34}$$

As a result of this construction, we can add an additional output coupler $(1 : n)$ to compute the transmission coefficient without modifying the lumped element circuit components in the model (Supplementary Fig. 2). We write the effective turns ratio of the input and output coupler as n_1 and n_2 respectively. Critical coupling of the input loop alone (with no output coupling) is achieved when $n_1 = \sqrt{Z_0/R}$, where Z_0 is the input line impedance (typically 50 Ω). The effective turns ratios can be controlled by changing the distance from the loop to the resonator.

B. Spin ensemble contribution to cavity response

To incorporate the spin ensemble's effect on the composite cavity response, consider the contribution of the spin-ensemble magnetization to the flux through the resonator. When a coil of inductance L is filled with a material of magnetic susceptibility χ_m, its inductance increases to $L(1+\chi_m)$ [7]. For the model investigated here, the complex susceptibility modifies the series inductance to $L_s(1+\chi'-j\chi'')$. Using the approximation that $\chi << 1$ the total admittance of the composite cavity can be written as

$$Z^{-1} = \left[\frac{1}{R} + \frac{\chi''}{\omega_d L}\right] + \left[\frac{1}{j\omega_d L}(1-\chi') + j\omega_d C\right]. \tag{35}$$

which shows the contribution of the absorption to the real part and the dispersion to the imaginary part of the impedance.

C. Reflection and transmission coefficients

We calculate the voltage reflection (Γ) and transmission (T) coefficients,

$$\Gamma = \frac{n_1^2 - n_2^2 - Z_0 Z^{-1}}{n_1^2 + n_2^2 + Z_0 Z^{-1}} \tag{36}$$

and

$$\mathrm{T} = \frac{2 n_1 n_2}{n_1^2 + n_2^2 + Z_0 Z^{-1}}, \tag{37}$$

for the circuit with input and output couplers using classical circuit theory [8]. Making the near-resonance approximation for the resonator gives

$$\Gamma = -1 + \frac{2n_1^2/Z_0}{1/R + j2C(\omega_d - \omega_c) + n_1^2/Z_0 + n_2^2/Z_0 - \chi(j\omega_d L)^{-1}} \tag{38}$$

and

$$T = \frac{2n_1 n_2/Z_0}{1/R + j2C(\omega_d - \omega_c) + n_1^2/Z_0 + n_2^2/Z_0 - \chi(jL\omega_d)^{-1}}. \tag{39}$$

To obtain the reflection and transmission coefficients used in the main text, we identify $\kappa_{c0} = 1/(RC)$, $\kappa_{c1} = n_1^2/(Z_0 C)$, and $\kappa_{c2} = n_2^2/(Z_0 C)$. Using Supplementary Eq. (9) with the approximation $\omega_d \approx \omega_c$ and the substitutions $\kappa_s = 2/T_2$ and $\kappa_{\mathrm{op}} = 1/T_1^{\mathrm{op}}$ and the relationship

$$\left(\gamma \frac{B_1^{\perp}}{2}\right)^2 = g_s^2 n_{\mathrm{cav}} \tag{40}$$

between the single-spin-photon coupling g_s and the driving magnetic field B_1, along with the relationship

$$g_{\mathrm{eff}}^2 = \frac{\chi_0}{4}\omega_c \omega_s \tag{41}$$

between the effective collective coupling g_{eff} and the static susceptibility χ_0, gives the reflection (1) and transmission (4) expressions in the main text. These are equivalent to those obtained through the circuit QED treatment [9, 10].

To incorporate the inhomogeneous distribution of spin resonance frequencies, we integrate the spin response over the appropriate probability density function $\rho(\Delta)$ where, for example, in the numerical model discussed in Supplementary Note 5 $\rho(\Delta)$ is given by a Gaussian probability density function. The full reflection and transmission coefficients

become

$$\Gamma = -1 + \frac{\kappa_{c1}}{\frac{\kappa_{c0}+\kappa_{c1}+\kappa_{c2}}{2} + j(\omega_d - \omega_c) + \int_{-\infty}^{\infty} \rho(\Delta) \left[\frac{g_{\text{eff}}^2}{\frac{\kappa_s}{2} + j(\omega_d - \omega_s - \Delta) + \frac{g_s^2 n_{\text{cav}} \kappa_s/(2\kappa_{op})}{\frac{\kappa_s}{2} - j(\omega_d - \omega_s - \Delta)}} \right] d\Delta} \tag{42}$$

$$T = \frac{\sqrt{\kappa_{c1}\kappa_{c2}}}{\frac{\kappa_{c0}+\kappa_{c1}+\kappa_{c2}}{2} + j(\omega_d - \omega_c) + \int_{-\infty}^{\infty} \rho(\Delta) \left[\frac{g_{\text{eff}}^2}{\frac{\kappa_s}{2} + j(\omega_d - \omega_s - \Delta) + \frac{g_s^2 n_{\text{cav}} \kappa_s/(2\kappa_{op})}{\frac{\kappa_s}{2} - j(\omega_d - \omega_s - \Delta)}} \right] d\Delta}. \tag{43}$$

We note that g_{eff}^2 in the above equations may be replaced by $g_s^2 N$ (see next section).

IV. SUPPLEMENTARY NOTE 4: COOPERATIVITY

A. Collective cooperativity ξ

The single-spin-photon coupling is

$$g_s = \frac{\gamma}{2} \mathfrak{n}_\perp B_v^{\text{RMS}}(\mathbf{r}), \tag{44}$$

where $B_v^{\text{RMS}}(\mathbf{r})$ is the RMS vacuum B-field in the cavity at the spin defect location $\mathbf{r}$ and $\mathfrak{n}_\perp$ denotes the projection of $\hat{B}_v^{\text{RMS}}$ onto a plane perpendicular to the NV axis (i.e., the component of $\mathbf{B}_v^{\text{RMS}}$ capable of driving a transition $|m_s = 0\rangle \to |m_s = \pm 1\rangle$). We take $\mathbf{B}_v^{\text{RMS}} \parallel \mathbf{B}_1$, where $\mathbf{B}_1$ is the magnetic field of the cavity-enhanced MW drive. The factor of $\frac{1}{2}$ results from a combination of the rotating wave approximation and the linear polarization of B_1 in the lab frame. For estimation purposes, we assume the B_1 field projects equally onto all four NV axes so that $\mathfrak{n}_\perp = \sqrt{\frac{2}{3}}$. Assuming the spins are located at the cavity antinode, which is a reasonable approximation for this geometry, we have

$$B_v^{\text{RMS}} = \sqrt{\frac{\hbar \omega_c \mu_0}{V_{\text{cav}}}}, \tag{45}$$

where V_{cav} is the cavity mode volume defined as

$$V_{\text{cav}} = \frac{\int |\mathbf{B}(\mathbf{r})|^2 dV}{|\mathbf{B}_{\text{max}}|^2}. \tag{46}$$

Then Supplementary Eq. (44) gives [11, 12]

$$g_s = \frac{\gamma}{2} \mathfrak{n}_\perp \sqrt{\frac{\hbar \omega_c \mu_0}{V_{\text{cav}}}}. \tag{47}$$

Based on finite element software modeling of the cavity, we find that $g_s/(2\pi) = 0.02 \pm 0.001$ Hz. When the number of cavity photons $n_{\text{cav}} \gg 1$, the Rabi frequency Ω_R can be approximated as

$$\Omega_R \approx \gamma \mathfrak{n}_\perp \sqrt{\frac{\hbar \omega_c \mu_0}{V_{\text{cav}}}} \sqrt{n_{\text{cav}}}. \tag{48}$$

Then we have

$$g_s = \frac{\Omega_R}{2\sqrt{n_{\text{cav}}}}. \tag{49}$$

The effective collective (ensemble) spin-photon coupling is given by $g_{\text{eff}} = g_s\sqrt{N}$ [13, 14] , where N is the number of polarized spins. From the data in Fig. 2 (in the main text), g_{eff} is found to be $2\pi \times 0.7$ MHz. Then we find $N \approx 1.4 \times 10^{15}$. Finally, the collective cooperativity is given by [15]

$$\xi = \frac{4 g_{\text{eff}}^2}{\kappa_c \kappa_s}, \tag{50}$$

where $\kappa_c = \omega_c/Q_L$ is the cavity loss rate and κ_s is the spin decoherence time (each in angular frequency units).

V. SUPPLEMENTARY NOTE 5: DISPERSIVE READOUT ANALYSIS

A. Optimizing the NV magnetometer

To optimize MW cavity readout for magnetometry, we investigate the spin-cavity system behavior at higher MW powers by plotting the reflection signal as a function of $\omega_s - \omega_c$ for a range of MW amplitudes (Supplementary Fig. 3a). For incident MW powers above -20 dBm the fractional absorption is suppressed, resulting in little reflected power on resonance. We use the slope $\partial V_{\mathrm{RMS}}/\partial(\omega_s - \omega_c)$ as a figure of merit to determine the optimal applied MW power for best signal-to-noise ratio, which, for the selected diamond, occurs at 10 dBm. As shown in Supplementary Fig. 3a, an increase in the applied MW power above 10 dBm yields a reduced slope due to excessive MW power broadening of the spin resonance. For magnetometry, the reflected signal is mixed to baseband using an in-phase and quadrature (IQ) mixer. Supplementary Fig. 3b plots a measured IQ mixer output (corresponding to 5 dBm incident MW power) where the dispersion effect on the cavity is isolated to the quadrature channel and the absorption effect is isolated to the in-phase channel. The maximum slope and consequently the greatest sensitivity occurs in the quadrature channel at $\omega_s = \omega_c$.

B. Numerical model

The need for a numerical model of our system (rather than an analytical model) arises primarily due to a single complexity: the line centers of individual NV$^-$ defects within the ensemble vary due to inhomogeneous strain. The presence of hyperfine structure can similarly be viewed as effectively corresponding to triplets of NV$^-$ defects with differing line centers. We find that these effects must be accounted for in our model to reproduce the experimental results; this is expected, given that the line-center variation is large compared to both $2/T_2$ and $1/T_1^{\mathrm{op}}$. When data is fit using the 2D nonlinear least-squares solver (e.g., for Fig. 2 in the main text), these effects are accounted for by interpreting $\kappa_s = 2/T_2$ in the reflection and transmission equations as an effective $\kappa_s^* = 2/T_2^*$ parameter including the effects of strain broadening and hyperfine structure.

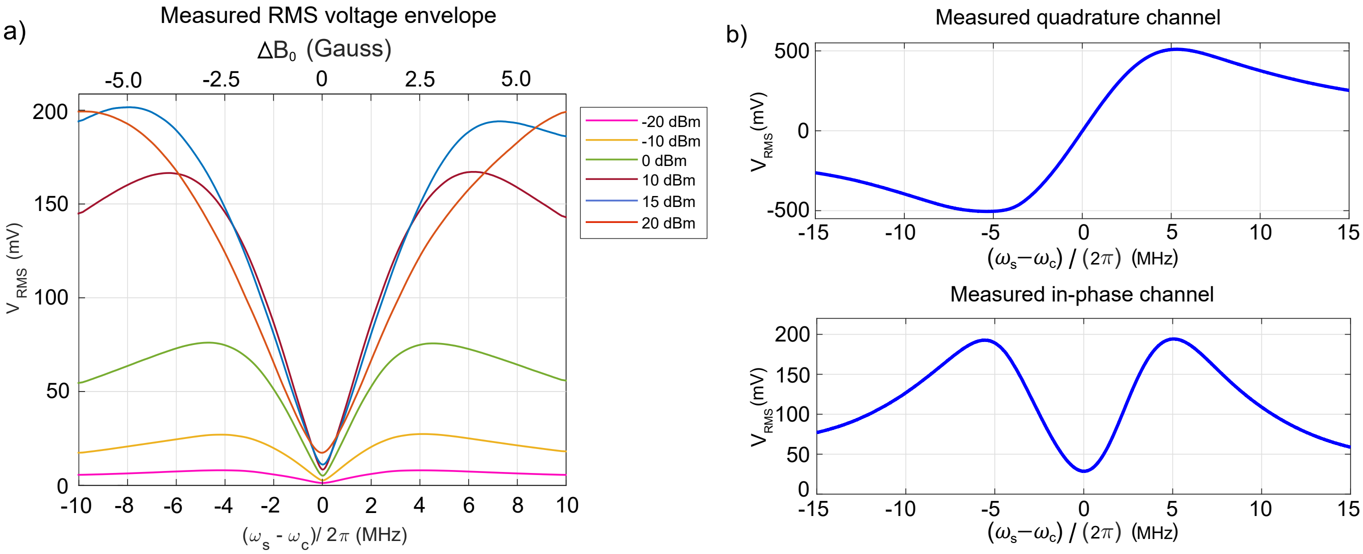

Supplementary Fig. 3. **Optimizing MW cavity readout for magnetometry.** a) The 2.901 GHz reflection signal is terminated into the 50 Ω input of a 40 GS/s oscilloscope. The reflected RMS voltage into 50 Ω is plotted vs the spin-cavity detuning for various MW powers. Above approximately 10 dBm, MW-induced broadening of the NV$^-$ ground state transition reduces the achievable magnetic sensitivity of the sensor; consequently 10 dBm is the near-optimal applied MW power. b) Measured in-phase and quadrature channels of an IQ mixer during MW cavity readout at 5 dBm of applied MW power. The MW drive ω_d is set to the bare cavity frequency ω_c, and, using the test coil, the spin-cavity detuning $(\omega_s - \omega_c)$ is swept from -15 MHz to 15 MHz. Finally, the phase of reference component (See Fig 1) is adjusted manually until the dispersive and absorptive signals are isolated to the quadrature and in-phase channels of the mixer, respectively. Data was taken under lower MW irradiation than in Figures 2-3 to avoid saturating the output of the low noise amplifier. The voltage signal here is measured after amplification and mixing with an effective gain (comprised of the amplifier gain 18 dB and mixer conversion loss 10.5 dB) of 7.5 dB.

In contrast, strain broadening and ^{14}N hyperfine structure are built into the full numerical model by integrating Supplementary Eq. (7) and Supplementary Eq. (8) over a distribution of line centers rather than a single resonance frequency. The strain broadening is approximated by a Gaussian probability density function

$$p_G(\delta\omega) = \frac{1}{\sqrt{2\pi\sigma_G^2}} e^{-\delta\omega^2/(2\sigma_G^2)} \tag{51}$$

where σ_G is the standard deviation and $\delta\omega$ denotes the deviation from the mean line center at ω_s. We achieve good agreement with experimental data using $\sigma_G = 2\pi \times 2.1$ MHz. The ^{14}N hyperfine are treated as a triplet with $2\pi \times 2.16$ MHz spacing between adjacent lines; the final line-center distribution is a convolution of the Gaussian strain distribution given by Supplementary Eq. (51) with the hyperfine triplet spectrum (where each hyperfine line is represented by a delta function).

The cavity is modeled as a series RLC circuit with the values given by Supplementary Eq. (30). The cavity inductance L_s is modified by the complex susceptibility; equations Supplementary Eq. (7) and Supplementary Eq. (8) are adapted by replacing ω_s with $\omega_s + \delta\omega$ and integrating with the line-center distribution described above. The total impedance of the cavity is calculated using Supplementary Eq. (35). Reflection and transmission are then calculated using Supplementary Eq. (36) and Supplementary Eq. (37).

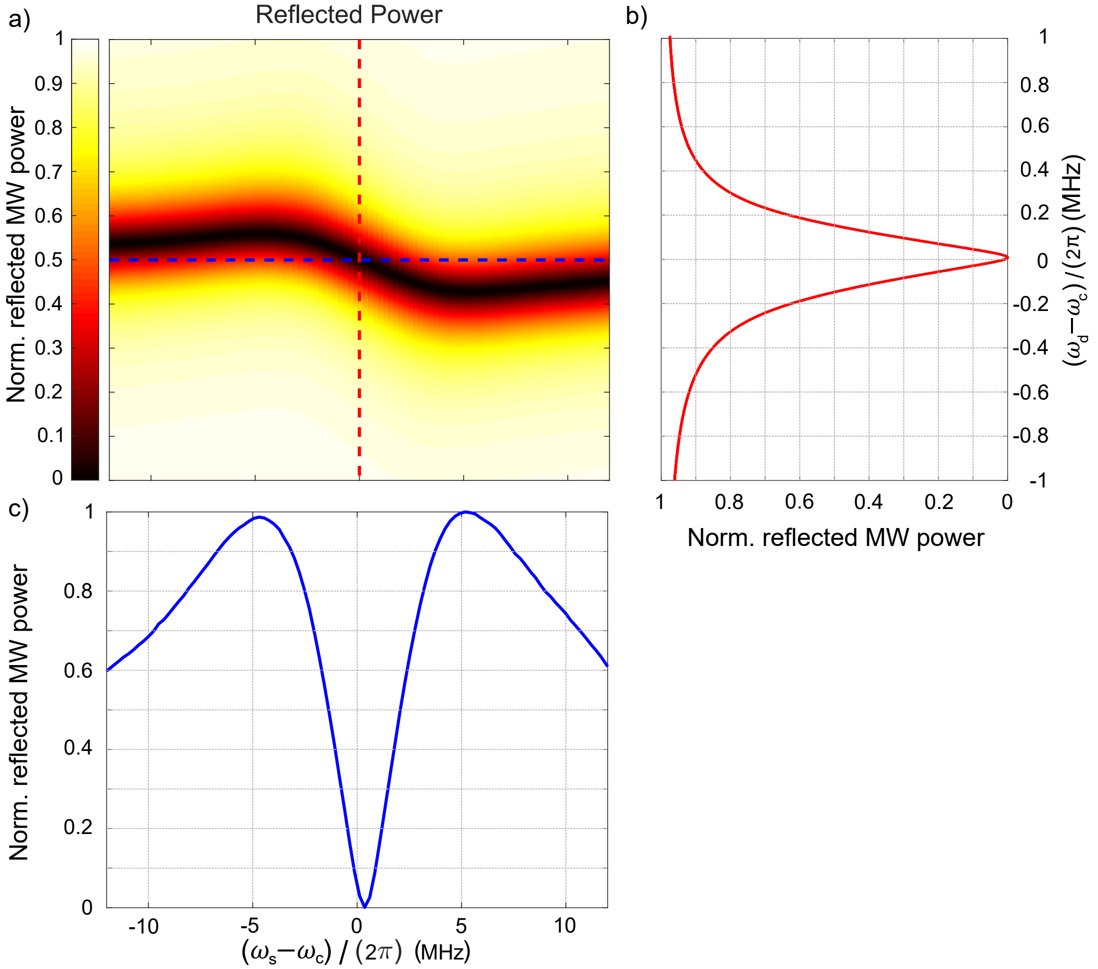

Supplementary Fig. 4. **2D MW cavity readout spectrum**. a) Data taken at 0 dBm of applied MW power. b) A red vertical cross section (solid red line) reveals the reflection spectrum of the composite cavity, i.e. in electrical engineering terminology, an S_{11} measurement of the composite resonator. c) A blue horizontal cross section (solid blue line) at $\omega_d - \omega_c = 0$ shows the reflected power as the spin resonance is tuned over the cavity resonance. In experiment, this curve is produced by setting the MW drive ω_d to the bare cavity resonance ω_c and changing the spin transition frequency ω_s. The slight offset of the minimum from zero detuning, visible in b) and c), is attributed to thermal drift between when the detuning was calibrated and when the measurements were taken.

The parameters are then tuned manually to match the data. The value of χ_0 is varied until the peak-to-peak dispersive shift in the numerical model matches that observed in the experiment for very low applied microwave power (below -50 dBm). Thereafter χ_0 is fixed. Next, the value of H_1 for a given input power is computed from the finite element software model for the composite cavity. The values of T_1^{op}, T_2, and the strain distribution are adjusted manually until acceptable agreement with data is reached, and these parameter values are then checked by comparing simulations to reflection data taken at a range of input powers (-20 dBm to 20 dBm in 5 dBm increments). Ultimately we achieve good agreement using $T_2 = 20$ μs, $T_1^{\mathrm{op}} = 500$ μs, $\mathfrak{n}_\perp \chi_0 = 2.3 \times 10^{-7}$ and the strain distribution detailed above.

[1] D. Budker and M. Romalis, Optical magnetometry, Nature Physics **3**, 227 (2007).

[2] J. F. Barry, J. M. Schloss, E. Bauch, M. J. Turner, C. A. Hart, L. M. Pham, and R. L. Walsworth, Sensitivity optimization for NV-diamond magnetometry, Rev. Mod. Phys. **92**, 015004 (2020).

[3] A. Abragam, *Principles of Nuclear Magnetism* (Oxford Univ. Press, 1961).

[4] D. Kajfez and P. Guillon, eds., *Dielectric Resonators* (Artech House, 1986).

[5] S. B. Cohn, Microwave bandpass filters containing high-Q dielectric resonators, IEEE Transactions on Microwave Theory and Techniques **16**, 218 (1968).

[6] C. J. Montgomery, R. H. Dicke, and E. M. Purcell, *MIT Radiation Laboratory Series: Principles of Microwave Circuits*, Vol. 8 (McGraw-Hill, 1948).

[7] C. Slichter, *Principles of Magnetic Resonance* (Springer Berlin Heidelberg, 1996).

[8] D. M. Pozar, *Microwave Engineering* (John Wiley & Sons, 2009).

[9] A. A. Clerk, M. H. Devoret, S. M. Girvin, F. Marquardt, and R. J. Schoelkopf, Introduction to quantum noise, measurement, and amplification, Reviews of Modern Physics **82**, 1155 (2010).

[10] S. M. Girvin, Circuit QED: superconducting qubits coupled to microwave photons, Quantum Machines: Measurement and Control of Engineered Quantum Systems **113**, 2 (2011).

[11] D. I. Schuster, A. P. Sears, E. Ginossar, L. Dicarlo, L. Frunzio, J. J. L. Morton, H. Wu, G. A. D. Briggs, B. B. Buckley, D. D. Awschalom, and R. J. Schoelkopf, High-cooperativity coupling of electron-spin ensembles to superconducting cavities, Phys. Rev. Lett. **105**, 140501 (2010).

[12] X. Zhang, C.-L. Zou, L. Jiang, and H. X. Tang, Strongly coupled magnons and cavity microwave photons, Phys. Rev. Lett. **113**, 156401 (2014).

[13] M. Tavis and F. W. Cummings, Exact solution for an n-molecule—radiation-field hamiltonian, Physical Review **170**, 379 (1968).

[14] A. F. Kockum, A. Miranowicz, S. De Liberato, S. Savasta, and F. Nori, Ultrastrong coupling between light and matter, Nature Reviews Physics **1**, 19 (2019).

[15] H. Tanji-Suzuki, I. D. Leroux, M. H. Schleier-Smith, M. Cetina, A. T. Grier, J. Simon, and V. Vuletić, Interaction between atomic ensembles and optical resonators, Advances in Atomic Molecular and Optical Physics **60**, 201 (2011).
